# Real-World Safety of Esketamine Nasal Spray: A Comprehensive Analysis of Esketamine and Respiratory Depression

**DOI:** 10.1093/ijnp/pyae058

**Published:** 2024-11-29

**Authors:** Craig Chepke, Richard Shelton, Gerard Sanacora, Teodora Doherty, Palina Tsytsik, Nancy Parker

**Affiliations:** Sandra & Leon Levine Psychiatry Residency Program at Atrium Health, Charlotte, North Carolina, USA; Excel Psychiatric Associates, Huntersville, North Carolina, USA; Department of Psychiatry and Behavioral Neurobiology, The University of Alabama at Birmingham, Birmingham, Alabama, USA; The Yale Depression Research Program, Yale University, New Haven, Connecticut, USA; Johnson & Johnson Innovative Medicine, Research & Development, Titusville, New Jersey, USA; Johnson & Johnson Innovative Medicine Austria, Vienna, Austria; Johnson & Johnson Innovative Medicine, Research & Development, Horsham, Pennsylvania, USA

**Keywords:** depression, esketamine nasal spray, ketamine, respiratory depression

## Abstract

**Background:**

Esketamine nasal spray (ESK) is approved, in conjunction with an oral antidepressant, for the treatment of treatment-resistant depression in adults and for the treatment of depressive symptoms in adults with major depressive disorder with acute suicidal ideation or behavior. No adverse events (AEs) of respiratory depression were reported in ESK phase 3 clinical trials; however, postmarketing incidents of respiratory depression associated with ESK use have been observed.

**Methods:**

The Janssen Global Medical Safety (GMS) database was reviewed for cases meeting the criteria for respiratory depression with ESK using 47 months of postapproval data, based on the Standardized Medical Dictionary for Regulatory Activities (MedDRA) Query (SMQ) acute central respiratory depression (broad). FDA Adverse Event Reporting System (FAERS), EudraVigilance, and literature searches were performed to identify reports of respiratory depression related to ESK use.

**Results:**

Fifty cases, representing 50 patients, in the GMS database met the case definition for respiratory depression; 8 of these had a stronger association with ESK use. The MedDRA preferred term (PT) hypopnea met the threshold for disproportionality with ESK in FAERS. The MedDRA PTs asphyxia, oxygen saturation decreased, respiratory depression, and apnea met the threshold for disproportionality with ESK in EudraVigilance.

**Conclusion:**

Despite extensive soliciting of AEs for ESK with the US Risk Evaluation and Mitigation Strategy program, respiratory depression is infrequently observed with ESK treatment in the postmarketing setting (estimated incidence: 1 case per 20 000 treatment sessions). Symptoms are manageable and resolve with minor supportive measures. Monitoring for symptoms of respiratory depression, including pulse oximetry, is recommended within the postdose observation period.

**Significance Statement**

Esketamine nasal spray (ESK) is a noncompetitive *N*-methyl-d-aspartate (NMDA) receptor antagonist approved, in conjunction with an oral antidepressant, for the treatment of treatment-resistant depression in adults and for the treatment of depressive symptoms in adults with major depressive disorder with acute suicidal ideation or behavior. Postmarketing incidents of respiratory depression associated with ESK use have been reported. This study was conducted to examine the real-world evidence of respiratory depression in association with ESK using 47 months of postapproval data collected by Janssen through spontaneous and stimulated reporting in the United States and globally. A cumulative analysis from the Janssen Global Medical Safety (GMS) database demonstrated that rare cases of respiratory depression were reported in association with ESK treatment in patients with comorbidities such as obesity, anxiety, and cardiovascular and respiratory conditions.

## INTRODUCTION

Esketamine nasal spray (ESK) was approved by the US Food and Drug Administration (FDA), in conjunction with an oral antidepressant, for the treatment of treatment-resistant depression in adults in March 2019 and for the treatment of depressive symptoms in adults with major depressive disorder with acute suicidal ideation or behavior in July 2020 (Janssen Pharmaceuticals, 2023). ESK is now approved in 78 countries worldwide. ESK can induce rapid antidepressant effects and is conveniently delivered as a nasal spray, allowing for self-administration by patients under the supervision of a health care provider (Janssen Pharmaceuticals Inc., 2024). From launch to January 31, 2023, the cumulative ESK exposure is estimated to be 77 868 person-years.

Ketamine and ESK are noncompetitive *N*-methyl-d-aspartate receptor antagonists and have been widely used to induce and maintain anesthesia. Both ketamine and ESK cause sedation, and although ketamine generally stimulates respiration, rapid administration may result in transient respiratory depression (Par Pharmaceutical, 2024). Maintenance of respiration is achieved through coordination and communication between the central nervous system and sensory mechanisms in the periphery, including the lungs and respiratory muscles (Webster and Karan, 2020). Some centrally acting drugs are known to suppress the regulation of respiration, leading to respiratory depression; these include opioid analgesics, barbiturates, benzodiazepines, other hypnotic sedatives, and ethanol, each with unique mechanisms of action to suppress respiration (Webster and Karan, 2020).

In the phase 3 clinical studies of ESK, decreases in oxygen saturation (SpO_2_) levels (reported in less than 3% of participants exposed to ESK) were not associated with clinical symptoms indicative of respiratory depression nor did they require resuscitation and were not considered clinically relevant; therefore, they were not reported as adverse events (AEs) by the investigators in the majority of cases (Daly et al., 2019; Fedgchin et al., 2019; Popova et al., 2019; Fu et al., 2020; Ionescu et al., 2020; Ochs-Ross et al., 2020; Wajs et al., 2020; Reif et al., 2023). However, postmarketing incidents of respiratory depression associated with ESK use have been reported. Based on this new safety information, product label changes regarding cases of respiratory depression with ESK have been approved by regulatory authorities. The objective of this study was to examine the real-world evidence of respiratory depression in association with ESK using 47 months of postapproval data collected by Janssen through spontaneous and stimulated reporting in the United States and globally, to describe patient characteristics, reporting rate and incidence of respiratory depression, and serious AEs of respiratory depression, including their resolution and outcome.

## METHODS

### GMS Global Safety Database

The Janssen Global Medical Safety (GMS) database gathers data on ESK use worldwide and compiles surveillance data on AEs reported from clinical trials, postmarketing surveillance studies, spontaneous reports, literature reports, regulatory authorities, reports on social media, and reports from the ESK Risk Evaluation and Mitigation Strategy (REMS) program. In the United States, ESK is only available through a restricted REMS program to evaluate and mitigate the risks of serious AEs resulting from sedation, dissociation, respiratory depression, abuse, and misuse (Janssen Pharmaceuticals, 2023). The REMS program solicits reports of AEs associated with ESK administration, along with the timing of onset of these events.

The GMS database was reviewed for cases meeting the following criteria: ESK as the suspect or suspect-interacting drug; health care professional (HCP)–reported cases; non-HCP–reported cases; all initial and follow-up reports and the most current version of cases in the specified date range (March 5, 2019 to February 8, 2023). Events indicative of respiratory depression were identified based on the Standardized Medical Dictionary for Regulatory Activities (MedDRA) Query (SMQ) acute central respiratory depression (broad). The specific focus was made on the cases reporting latency to onset of less than 120 minutes, positive rechallenge (reoccurrence of the same AE when the treatment is resumed after interruption), stand-alone respiratory symptoms or abnormal respiratory values, specifically, SpO_2_ of ≤93% and/or respiratory rate (RR) of ≤10 breaths per minute (bpm).

### FDA Adverse Event Reporting System and EudraVigilance Data

A search of the FDA Adverse Event Reporting System (FAERS) was conducted to identify case counts and empirical Bayesian geometric mean (EBGM) scores for reports of the MedDRA SMQ acute central respiratory depression (broad) for ESK and ketamine (all routes of administration). A drug-event combination (DEC) was identified as higher than expected (disproportionately reported) within a database if there was an EBGM of ≥2 and a case count of ≥3. An EBGM of 2 represents a 2-fold increase in reporting of a DEC as compared with all other drugs in the database. A search of the EudraVigilance safety database was conducted to obtain case counts and values for the lower limit of the 95% confidence interval for the reporting odds ratio (ROR 025), with DECs with disproportional reporting identified with a ROR 025 >1, and a case count ≥3 (for ESK) or ≥5 (for ketamine).

### Literature

A cumulative literature search for ESK and respiratory depression was performed through March 5, 2023, for all reports/articles relating to the use of esketamine. The biomedical databases searched included Ovid MEDLINE^®^ ALL (1946 to March 7, 2023) and Embase (1974 to March 7, 2023). Additionally, 9 publications issued by the FDA were screened for relevant safety information.

## RESULTS

### GMS Global Safety Database

**Figure 1.**
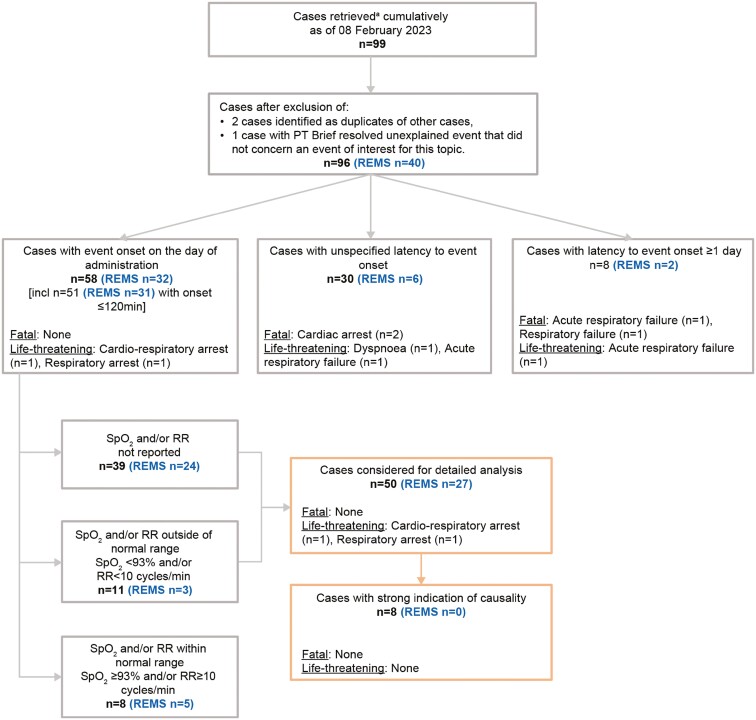
Case screening flowchart for the GMS Global Safety Database. GMS, Global Medical Safety; MedDRA, Medical Dictionary for Regulatory Activities; PT, preferred term; REMS, Risk Evaluation and Mitigation Strategy; RR, respiratory rate; SMQ, standard MedDRA query; SpO_2_, oxygen saturation. ^a^Search based on MedDRA SMQ acute central respiratory depression (broad).

The search of the GMS global safety database retrieved 96 cases after the exclusion of irrelevant cases (eg, duplicates); of these, detailed evaluation was conducted for 50 cases that met the case definition for respiratory depression, namely plausible latency with onset on the day of ESK administration and respiratory values either outside of the normal range (SpO_2_ ≤93% and/or RR of ≤10 bpm) or not reported according to the case screening strategy (Figure 1). Case characteristics are shown in Table 1.

**Table 1.** Case Characteristics for Cases Found in the GMS Global Safety Database.

Characteristic		Number of Cases *N* = 50
**Country/territory of origin**	United States of America	35
	France	6
	Costa Rica	2
	Republic of Korea	2
	Germany	1
	Israel	1
	Canada	1
	Monaco	1
	United Kingdom	1
**Source**	REMS and other solicited sources	31
	Spontaneous	17
	Non-interventional studies	2
	Interventional clinical trials	0
**Time to onset from recent dose**	≤120 minutes	43
	>120 minutes, the same day	7
**Latest dose before event(s) onset, mg**	84	21
	56	17
	28	1
	NR	11
**Intervention for the event(s)**	EMS/ER visit/hospitalization	12
	Oxygen	9
	Stimulation	4
	Medication	4
	None	3
	Rescue breathing and/or CPR	2
	Not otherwise specified	1
	NR	15
**Reported SpO**_**2**_ **and RR**	SpO_2_ ≤93% and/or RR ≤10 bpm	11
	NR	39

Abbreviations: CPR, cardiopulmonary resuscitation; EMS/ER, emergency medical services/emergency room; ESK, esketamine nasal spray; GMS, Global Medical Safety; NR, not reported; REMS, Risk Evaluation and Mitigation Strategy; RR, respiratory rate; SpO_2_, oxygen saturation.

Fifty patients were represented within these 50 cases (Table 2). The majority of patients were female (*n* = 31, 62.0%), consistent with the ratio receiving ESK treatment. The indications for ESK treatment were available for 40 patients, and depression (38/40) was the most common indication reported, although multiple indications could be reported per patient. Concomitant medications that could have equally caused or contributed to respiratory depression were reported in 36/50 patients, with the most frequently reported being medications from the drug classes of benzodiazepines (18/36) and antidepressants (16/36). The most commonly reported relevant comorbidities that could have contributed or predisposed to respiratory depression were anxiety/panic disorder/posttraumatic stress disorder (*n* = 23), obesity (*n* = 8), cardiovascular disease (*n* = 4), and respiratory conditions (*n* = 3). An unhealthy lifestyle was reported in 4 cases.

**Table 2.** Patient Characteristics for Cases Found in the GMS Global Safety Database.

Characteristic		Number of Patients *N* = 50
**Sex**	Female	31
	Male	17
	NR	2
**Age,** ^a^ **years**	18 to 35	10
	36 to 50	13
	51 to 64	17
	≥65	4
	NR	6
**Indication** ^b^	Depression	38
	Major depression	4
	Suicidal ideation	1
	Depression suicidal	1
	Mental disorder	1
	NR	10
**Relevant concomitant medications by drug category** ^c^	Benzodiazepine	18
	Antidepressant	16
	SNRI/SSRI	7
	None	9
	Hypnotic	4
	Antipsychotic	4
	Anticonvulsant	4
	Beta blocker	2
	Antihistamines	1
	NR	14
**Comorbidities** ^d^	Anxiety/panic disorder/PTSD	23
	None	11
	Obesity	8
	Cardiovascular disease	4
	Unhealthy lifestyle	4
	Asthma	2
	COPD	1
	NR	11

^a^For cases where age is not reported, reported age category is presented.

^b^Multiple indications could be reported per patient. This information was available for 40 out of 50 patients (80%).

^c^Multiple concomitant medications could be reported per patient. This information was available for 36 out of 50 patients (72%).

^d^Multiple comorbidities could be reported per patient. This information was available for 39 out of 50 patients (78%).

Abbreviations: COPD, chronic obstructive pulmonary disease; GMS, Global Medical Safety; NR, not reported; PTSD, posttraumatic stress disorder; SNRI/SSRI, serotonin–norepinephrine reuptake inhibitor/selective serotonin reuptake inhibitor.

A summary of all AEs related to respiratory depression (*n* = 51) reported in these cases is presented in Table 3. The most common MedDRA preferred terms (PTs) were dyspnea (*n* = 24), oxygen saturation decreased (*n* = 8), and RR decreased (*n* = 3)—the remaining PTs occurred in ≤2 patients each. The majority (35/51) of events were serious.

**Table 3.** Event Characteristics for Cases Found in the GMS Global Safety Database.

Characteristic		Number of Events *N* = 51
**MedDRA PTs**	Dyspnea	24
	Oxygen saturation decreased	8
	Respiratory rate decreased	3
	Hypopnea	2
	Hypoxia	2
	Respiratory depression	2
	Respiratory distress	2
	Respiratory arrest	2
	Oxygen saturation abnormal	2
	Apnea	1
	Cardiorespiratory arrest	1
	Respiratory disorder	1
	Respiration abnormal	1
**Event seriousness**	Serious	35
	Nonserious	16
**Outcome and time to resolution**	Resolved in ≤120 minutes	15
	Resolved in >120 minutes	11
	Resolved, time unspecified	1
	Resolving	3
	Not resolved	2
	NR	19

Abbreviations: GMS, Global Medical Safety; MedDRA, Medical Dictionary for Regulatory Activities; NR, not reported; PT, preferred term.

Of the 50 cases meeting the case definition described above, 43 (86%) were reported with the latency to event onset of 120 minutes or less from ESK administration; that is, within the patient postdose observation period prescribed by the current guideline on ESK use. In the remaining 14% of cases, the events were reported to occur on the day of ESK administration, but the time of onset was not specified.

In 29 cases (58%), events were reported as resolved or resolving. In 19 cases, the outcome of the events was unknown, and in 2 cases, the patients had not recovered from events at the time of reporting. One case referred to nonserious dyspnea as a manifestation of a panic reaction along with other symptoms, which did not abate after the patient left the treatment center. The other case referred to dyspnea in conjunction with chest pressure and migraine headache, which did not abate within 120 minutes of observation and resulted in hospitalization for diagnostic clarification. At this time, no further information is available regarding the 2 cases with events of interest that were reported as not resolved.

In 15 cases, events were easily reversible and resolved within 120 minutes of observation. In 12 cases, patients required prolonged monitoring and/or involvement of either emergency medical services or an emergency department visit. In 6 of these cases, the events resulted in hospitalization, with duration either unknown (5 cases) or <1 day (1 case). Further case-level review of the reported hospitalizations suggested referral for diagnostic clarification of the patient’s clinical status due to other accompanying symptoms such as chest pain (3 cases), blood pressure increase (2 cases), or anxiety (1 case).

None of the 50 cases selected for closer analysis were fatal. Two cases were reported as life-threatening, referring to respiratory arrest and cardiorespiratory arrest, respectively. Both of these occurred in the context of sedation and/or dissociation during the treatment session, and in both cases, patients’ medical history and multiple concomitant medications suggested additional factors that could have contributed or predisposed to the reported events. One additional case coded with MedDRA PT respiratory arrest was not considered life-threatening. In all 3 of these cases, the patients recovered from the events.

The first patient with a life-threatening event became unresponsive, “was not breathing” (coded with MedDRA PTs unresponsive to stimuli, respiratory arrest), and was given rescue breathing, then responded and became alert. The patient was discharged after an observation period of 150 minutes. The patient’s history of polysubstance abuse and kidney transplant for end-stage renal disease may have contributed to the events. The second patient with a life-threatening event became unresponsive, looked like they were not breathing, and had no pulse (coded with MedDRA PT cardiorespiratory arrest). Cardiopulmonary resuscitation was performed, and the patient’s pulse and respirations restarted. The patient was taken to the emergency department and released on the same day. The patient had a history of syncope, very low blood pressure, hypothyroidism, and of note, used a cardiac loop recorder, indicating a possible cardiac pathology under consideration. Concomitant medication use of lithium, clonazepam, eszopiclone, trazodone, and bupropion might have contributed to the events.

In the additional serious case that was not considered life-threatening, the patient was reported to have “paralysis” and “stopped breathing” (coded with MedDRA PTs paralysis, respiratory arrest) along with visual hallucination and autoscopy; the patient resumed normal breathing after being seated upright by a nurse. The symptoms appear more suggestive of a dissociative state and in the absence of key information including the vital signs, relevant medical history, and concomitant medication, a comprehensive medical assessment is not possible.

In 25 cases (50%), symptoms indicative of respiratory depression were co-reported with sedation and dissociation. Current guidance for ESK use mandates close patient monitoring for at least 120 minutes to manage events of sedation and dissociation and serious outcomes that may also include respiratory depression.

Overall, a strong indication of causal association was present in 8 cases, representing 8 unique patients, all reported outside the United States. These cases met at least 3 of the 4 criteria for causality, namely latency to onset ≤120 minutes, positive rechallenge, stand-alone respiratory symptoms, or respiratory depression confirmed by reported respiratory values. The events were reported as resolved in 5 cases. In the remaining 3 cases, the outcome of events was reported as unknown.

No consistent pattern in the onset of SpO_2_ decrease was identified. In 5 patients, symptoms reappeared after readministration of ESK (positive rechallenge); in 2 of these patients, rechallenge reversed (ie, there was no further evidence of respiratory depression) after discontinuation of benzodiazepines (Rigoni et al., 2022). In the other 3 patients with positive rechallenge, no clinical symptoms were reported, and respiratory depression was diagnosed solely based on SpO_2_ measurements, with the lowest values of 85%, 85%, and 92%, respectively. Overall, events required only minor intervention, such as oxygen (in 3 patients), verbal or tactile stimulation (in 2 patients), or no intervention at all (in 2 patients); no information was provided for 1 patient.

### REMS Program

Forty of the 96 global cases of respiratory depression were reported from the US ESK REMS program, based on data collected from 868 446 treatment sessions from March 2019 to February 2023. Twenty-seven of these cases met the case definition, and none of these cases met the criteria of having a strong association with ESK (at least 3 of the following: latency to onset ≤120 minutes, positive rechallenge, stand-alone respiratory symptoms, or respiratory depression confirmed by reported respiratory values). Considering that REMS programs extensively stimulate the reporting of AE information and enable known patient exposure, the REMS data provide the best basis for estimating the incidence of respiratory depression in patients treated with ESK. Based on the data available as of February 2023, the incidence can be estimated at 1 case of respiratory depression per 20 000 treatment sessions.

### FAERS and EudraVigilance

Data mining revealed that the MedDRA PT hypopnea met the threshold for disproportionality with ESK in FAERS, with an EBGM score of 2.891. The MedDRA PTs asphyxia, oxygen saturation decreased, respiratory depression, and apnea met the threshold for disproportionality with ESK in EudraVigilance, with ROR 025 values of 1.93, 1.92, 1.27, and 1.26, respectively. The results from FAERS and EudraVigilance support the hypothesis that hypopnea, oxygen saturation decreased, respiratory depression, asphyxia, and apnea are statistically associated with ESK administration.

### Literature

A review of the literature revealed 1 citation deemed relevant to the research question. This preclinical study investigated the mechanism of respiratory depression in a µ-opioid knockout mouse model with *S*(+)-ketamine (Sarton et al., 2001). *S*(+)-ketamine (0, 10, 100, and 200 mg/kg) caused dose-dependent respiratory depression. At high doses (200 mg/kg), a reduction in the hypercapnic ventilatory response was observed. Greater respiratory depression and antinociception effects were observed in wild-type mice relative to µ-opioid knockout mice. The results suggest an interaction between *S*(+)-ketamine and the µ-opioid receptor system, leading to respiratory depression. However, extrapolation of the results to the human population requires further validation.

## DISCUSSION

The key safety findings and patient characteristics related to respiratory depression with the use of ESK globally suggest that the most frequently reported respiratory symptom is dyspnea, followed by oxygen saturation decreased. The majority (35/51) of the events were reported as serious. In most cases indicative of respiratory depression, symptoms appeared within 120 minutes of ESK administration, allowing for timely intervention within the mandated postdose observation period. Reports associated with symptoms indicative of respiratory depression have a low frequency of occurrence in the ESK postmarketing setting with an estimated incidence of 1 reported case per 20 000 treatment sessions.

Most of the cases of respiratory depression reported concurrent use of other central nervous system depressants and/or comorbidities such as anxiety, obesity, and cardiovascular and respiratory conditions. Temporal association supportive of causal association was reported in the majority of instances. Most symptoms resolved with supportive care, usually with minor supportive measures such as verbal or tactile stimulation or the administration of supplemental oxygen. There were no clinical manifestations of significantly depressed respiratory function and no fatal or life-threatening cases with strong indication of causality with ESK. Cases of respiratory arrest (2 patients) and cardiorespiratory arrest (1 patient), which occurred within 120 minutes of ESK administration, were reported in the context of deep sedation and/or dissociation, and all 3 patients recovered from the events following intervention measures provided within the observation period. In all cases, patients’ medical history and multiple concomitant medications suggested additional factors that could have contributed or predisposed to the reported events.

Data mining of postapproval AE reports is a valuable tool to detect potential safety signals using real-world evidence. Within the ESK primary data mining, hypopnea was disproportionately reported (ie, a ≥2-fold increase in reporting compared with all drugs in the database combined) in FAERS, and 4 MedDRA PTs (asphyxia, oxygen saturation decreased, respiratory depression, and apnea) were disproportionately reported in EudraVigilance. The results from FAERS and EudraVigilance support the hypothesis that there is a statistical association between ESK and these events.

Mitigation strategies and interventions for AEs observed following ESK administration are important to minimize risk to patients. For example, currently, dissociation, sedation, and blood pressure should be monitored for at least 120 minutes after ESK treatment, since these symptoms tend to peak around 40 minutes after dosing and typically resolve 90 minutes after dosing (Fedgchin et al., 2019; Janssen Pharmaceuticals, 2023). Notably, the occurrence of respiratory depression is significantly less frequent than that of hypertensive cases requiring intervention. The required mitigation strategies for respiratory depression depend on the regulatory region. In the United States, patients must be monitored for changes in respiratory status by an HCP for at least 120 minutes after ESK administration, including pulse oximetry, followed by an assessment to determine when the patient is considered clinically stable and ready to leave the health care setting.

The limitations of postmarketing surveillance are well known; voluntary reporting of individuals and HCPs is known to be subject to multiple forms of bias. These biases include the length of time a product has been on the market, country/territory, reporting environment, and quality of the data. Important limitations of spontaneous safety databases (company and regulatory databases) include general underreporting of postmarketing events and reporting biases. Due to the granularity of the MedDRA coding dictionary, the use of PTs for data mining can potentially obscure drug-event associations. The numerator and denominator data reflect only reports in the database, and the actual number of patients exposed to the products generally is not known, so it is not possible to calculate the true incidence of an AE from the database. Additionally, most products included in the FAERS database do not have a REMS program enriching the reporting of AEs, so direct comparison of AE rates experienced with ESK with alternative treatments is not possible. Furthermore, the FDA may recode PTs before cases are entered into FAERS and may also recode PTs as warranted at any time subsequent to entry. Therefore, it is important to consider medically similar terms in signal detection. Data mining is also limited by the absence of case-level details including medical history, details on dosing and administration, and the clinical course of the event.

In conclusion, respiratory depression manifesting predominantly as dyspnea may be observed with ESK treatment. The symptoms indicative of respiratory depression are reported very infrequently in the postmarketing setting, despite extensive soliciting of AEs for ESK. They are manageable and resolve with minor supportive measures. Monitoring for respiratory depression that includes pulse oximetry is recommended within the postdose observation period.

## Data Availability

The data sharing policy of Janssen Pharmaceutical Companies of Johnson & Johnson is available at https://www.janssen.com/clinical-trials/transparency. As noted on this site, requests for access to the study data can be submitted through Yale Open Data Access (YODA) Project site at http://yoda.yale.edu.

## Interest Statement

C.C. has served as a consultant for AbbVie, Acadia, Alkermes, Axsome, Biogen, Boehringer Ingelheim, Corium, Intra-Cellular, Janssen, Lundbeck, MedinCell, Moderna, Neurocrine, Noven, Otsuka, Sage, Sumitomo, Supernus, and Teva; has served on advisory boards for AbbVie, Acadia, Alkermes, Axsome, Biogen, Bristol Myers Squibb, Corium, Idorsia, Intra-Cellular, Janssen, Lundbeck, Moderna, Neurocrine, Noven, Otsuka, Sage, Sumitomo, and Teva; advisory board (spouse): Karuna and Otsuka; has served on speaker’s bureaus for AbbVie, Acadia, Alkermes, Axsome, Bristol Myers Squibb, Corium, Intra-Cellular, Janssen, Lundbeck, Merck, Neurocrine, Noven, Otsuka, Sumitomo, and Teva; and has received research and/or grant support from Acadia, Axsome, Harmony, Neurocrine, and Teva. R.S. has served as a consultant for Boehringer Ingelheim, Denovo Biopharma, Evecxia Therapeutics LLC, Johnson & Johnson Innovative Medicine, NeuroRx, Novartis AG, Otsuka Pharmaceuticals, Seelos Therapeutics, Inc., Sumitomo Group and Supernus Pharmaceuticals; has received research and/or grant support from National Institutes of Mental Health, Patient Centered Outcomes Research Institute, AbbVie Inc., Alto Pharmaceuticals, Boehringer Ingelheim, Denovo Biopharma, INmune Bio, Intra-cellular Therapies, Johnson & Johnson Innovative Medicine, LivaNova PLC, Navitor Pharmaceuticals, Neumora, Neurocrine Biosciences, NeuroRx, Novartis AG, Otsuka Pharmaceuticals, Sumitomo Group and Supernus Pharmaceuticals; and has received royalties from Springer-Nature Group and Wolters-Kluwer NV. G.S. has served as a consultant for Allergan, Alkermes, AstraZeneca, Avanir Pharmaceuticals, Axsome Therapeutics, Biohaven Pharmaceuticals, Boehringer Ingelheim International GmbH, Bristol Myers Squibb, Clexio, Denovo Biopharma, Engrail Therapeutics, Gilgamesh, Hoffmann-La Roche, Intra-Cellular Therapies, Janssen, Lundbeck, Merck, Naurex, Navitor Pharmaceuticals, Neurocrine, Novartis, Noven Pharmaceuticals, Otsuka, Praxis Therapeutics, Sage Pharmaceuticals, Servier Pharmaceuticals, Taisho Pharmaceuticals, Teva, Valeant, Vistagen Therapeutics, and XW Labs; has received research contracts from AstraZeneca, Bristol Myers Squibb, Eli Lilly and Company, Johnson & Johnson, Hoffmann-La Roche, Merck, Naurex, Servier, and Usona; holds equity in Biohaven Pharmaceuticals; and is a coinventor on a US patent (#8,778,979) held by Yale University and a coinventor on US provisional patent application no. 047162-7177P1 (00754) filed on August 20, 2018, by Yale University Office of Cooperative Research. Yale University has a financial relationship with Janssen Pharmaceuticals and may receive financial benefits from this relationship. The university has put multiple measures in place to mitigate this institutional conflict of interest. Questions about the details of these measures should be directed to Yale University’s Conflict of Interest office. T.D., P.T., and N.P. are employees of Janssen Research & Development, LLC, and may hold stock or restricted stock units in Johnson & Johnson, Inc.
